# Temporal echocardiographic assessment of pulmonary hypertension in idiopathic pulmonary fibrosis patients treated with nintedanib with or without oxygen therapy

**DOI:** 10.1186/s12890-019-0918-3

**Published:** 2019-08-22

**Authors:** Masahiro Tahara, Keishi Oda, Kei Yamasaki, Takako Kawaguchi, Konomi Sennari, Shingo Noguchi, Noriho Sakamoto, Toshinori Kawanami, Hiroshi Mukae, Kazuhiro Yatera

**Affiliations:** 1 Department of Respiratory Medicine, University of Occupational and Environmental Health, Japan, 1-1 Iseigaoka, Yahatanishiku, Kitakyushu city, Fukuoka, 807-8555 Japan; 2 Department of Respiratory Medicine, Nagasaki University Graduate School of Biomedical Sciences, 1-7-1 Sakamoto, Nagasaki city, Nagasaki, 852-8501 Japan

**Keywords:** Echocardiography, Idiopathic pulmonary fibrosis, Long-term oxygen therapy, Nintedanib, Pulmonary hypertension

## Abstract

**Background:**

Nintedanib is an inhibitor of receptor tyrosine kinases, including vascular endothelial growth factor receptor, but its effects on pulmonary hypertension (PH) in idiopathic pulmonary fibrosis (IPF) patients with chronic hypoxia were unclear.

**Methods:**

This study included a nintedanib prospective study and historical control study. In the nintedanib prospective study, pulmonary artery systolic pressure (PASP) measured using transthoracic echocardiography was evaluated at six points during 48 weeks in 16 IPF patients in whom nintedanib was started. In the historical control study, adjusted annual change in PASP was compared between patients treated with (*n* = 16) and without (*n* = 15) nintedanib.

**Results:**

In the nintedanib prospective study, the mean PASP at 48 weeks after starting nintedanib was significantly higher compared to that at baseline. When IPF patients were divided into two groups, IPF patients with or without long-term oxygen treatment (LTOT), mean PASP at 48 weeks was significantly higher than that at baseline only in IPF patients receiving LTOT (*P* = 0.001). In the historical control study, adjusted annual change in PASP in IPF patients treated with nintedanib was significantly lower than that in patients treated with no antifibrotic agents when considering patients without LTOT (0.26 mmHg vs 7.05 mmHg; *P* = 0.011).

**Conclusions:**

We found differential effects of nintedanib on PH between IPF patients with or without LTOT. Nintedanib may have a disadvantageous effect on PH in IPF patients with LTOT. Conversely, nintedanib treatment may be beneficial to PH in IPF patients without LTOT.

**Electronic supplementary material:**

The online version of this article (10.1186/s12890-019-0918-3) contains supplementary material, which is available to authorized users.

## Background

IPF is a fatal lung disease characterized by progressive loss of lung function, dyspnea, and cough [1]. Pulmonary hypertension (PH) is frequently found in IPF patients and is associated with an increase in the risk of death [2, 3].

Nintedanib (BIBF 1120) is an inhibitor of receptor tyrosine kinases, including vascular endothelial growth factor (VEGF) receptor, fibroblast growth factor (FGF) receptor, platelet-derived growth factor (PDGF) receptor, and non-receptor tyrosine kinases of the Src family [4]. These tyrosine kinases are involved in signaling pathways implicated in the development and progression of fibrosis [5]. The efficacy of nintedanib in idiopathic pulmonary fibrosis (IPF) patients was shown in the IMPULSIS trials and nintedanib has been approved for the treatment of IPF in several countries, including Japan [6].

In a hypoxic murine model using Sugen 5416 (SU5416), an inhibitor of the VEGF receptor, PH was exacerbated relative to hypoxic mice without SU5416 treatment [7]. Therefore, it is speculated that nintedanib, which inhibits the VEGF receptor, may adversely affect PH in IPF patients exposed to chronic hypoxia.

To evaluate the effect of nintedanib treatment on PH in IPF patients with or without oxygen therapy, this investigation included a nintedanib prospective cohort study and a historical control study, including 16 consecutive IPF patients in whom nintedanib treatment was started and 15 IPF patients without any antifibrotic therapies, respectively.

## Methods

### Patient population

IPF was diagnosed after multidisciplinary discussions in accordance with the criteria outlined by the 2011 consensus statements of the American Thoracic Society, European Respiratory Society, Japanese Respiratory Society, and Latin American Thoracic Association [8]. Among patients in the IPF cohort of the Department of Respiratory Medicine, University of Occupational and Environmental Health, Japan, between November 2015 and September 2017, IPF patients who started nintedanib treatment were enrolled in the nintedanib prospective cohort study. This prospective study was approved by the Institutional Review Board and Ethics Committee of University of Occupational and Environmental Health (IRB no: H27–180, UMIN: 000020392), and all patients provided written informed consent. We conducted a historical control study because patients with IPF usually experience the development of PH during their natural course. To match sample sizes, we used a historical control group comprising IPF patients who met the (a) and (b) criteria below from August 2008 to March 2016, retrospectively, (a) IPF patients who did not receive any antifibrotic therapies, and (b) IPF patients in whom echocardiography was performed at intervals of approximately 48 weeks. This retrospective study was approved by the Institutional Review Board and Ethics Committee of University of Occupational and Environmental Health, Japan (IRB no: 18–012).

### Study design

The following characteristics were assessed at baseline: age, sex, weight, body mass index, smoking status, time since the diagnosis of IPF, long-term oxygen treatment (LTOT), arterial oxygen saturation measured by pulse oximetry (SpO_2_) on room air at rest, partial pressure of arterial oxygen (PaO_2_) on room air at rest, results of the pulmonary function test (forced vital capacity [FVC], forced expiratory volume in 1 s [FEV_1_], and diffusing capacity of the lung for carbon monoxide [DL_CO_]), laboratory findings (brain natriuretic peptide [BNP]), radiographic findings (pulmonary emphysema), and comorbidities (hypertension, chronic heart failure). Dyspnea was assessed using the five-grade modified Medical Research Council (mMRC) dyspnea scale [9]. The Gender-Age-Physiology (GAP) score for IPF was also calculated in each patient, in accordance with the previously described methods [10].

In the nintedanib prospective study, pulmonary artery systolic pressure (PASP) measured using transthoracic echocardiography (TTE) and the results of pulmonary function test were evaluated at six periods (baseline, and 1, 4, 12, 24, and 48 weeks after starting nintedanib treatment). In the historical control study, PASP data were retrospectively collected, and annual change in PASP was adjusted to 48 weeks.

### Transthoracic echocardiography

Two-dimensional TTE using color Doppler flow method was performed, including measurement of the peak tricuspid regurgitation (TR) velocity. Right atrial pressure (RAP) was estimated on the basis of the inferior vena cava size and movement with respiration, and PASP was calculated using the modified Bernoulli equation (PASP = 4 × TR^2^ + RAP) [11, 12].

### Statistical analysis

Data are expressed as the median [range] or number of patients (percentage). Chi-square (χ^2^) tests were used to compare categorical variables. Comparisons between groups were performed using the Mann-Whitney *U* test. Differences in PASP and FVC between baseline and assessment after 48 weeks were evaluated using a paired t-test in the nintedanib prospective study. Adjusted annual changes in PASP were assessed using a two-sample t-test in the historical control study. *P* < 0.05 was considered significant. All analyses were performed using SPSS version 25 (SPSS, Inc.; Chicago, IL). MT and KO take complete responsibility for the integrity of the data and accuracy of the data analysis.

## Results

### The nintedanib prospective study

**Fig. 1:**
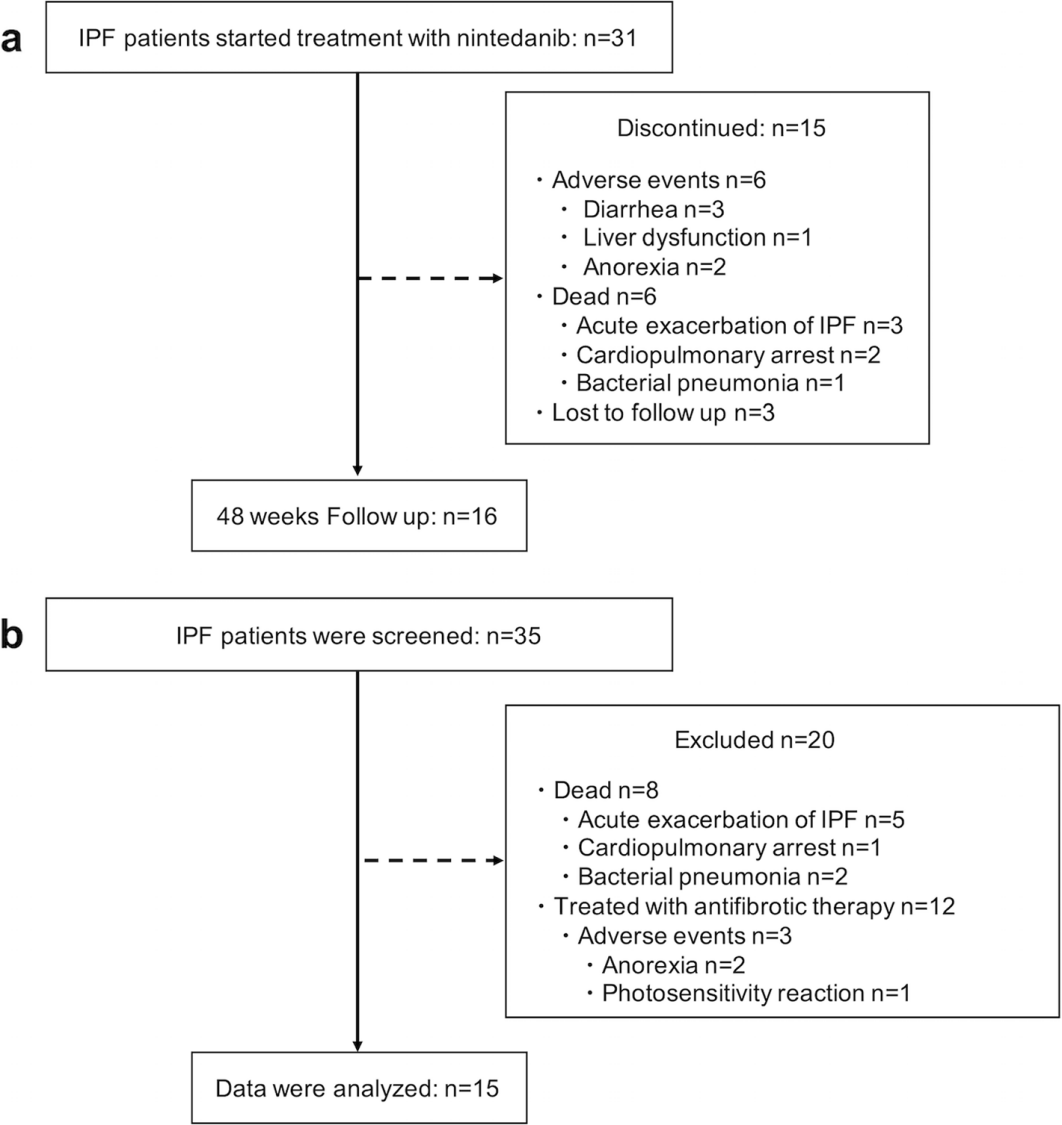
Patient flow diagrams of nintedanib prospective and historical control studies. **a** In the nintedanib prospective study, 31 consecutive idiopathic pulmonary fibrosis (IPF) patients were enrolled. Fifteen patients failed to continue nintedanib treatment for 48 weeks for various reasons (adverse events, *n* = 6; death, n = 6; lost to follow-up, *n* = 3). Follow-up studies were available in 16 patients at 48 weeks. **b** In the historical control study, 35 consecutive IPF patients were screened retrospectively. Of these patients, 20 IPF patients were excluded for some reason (poor quality echocardiography, *n* = 8; treated with anti-fibrotic therapy, *n* = 12). Finally, 15 IPF patients who did not receive any anti-fibrotic therapies were analyzed

Patient flow schematics of the nintedanib prospective study are shown in Fig. 1a. In the nintedanib prospective study, 31 consecutive IPF patients were enrolled, of which 15 patients failed to continue nintedanib treatment for 48 weeks, resulting in 16 patients available for the follow-up studies at 48 weeks (Fig. 1a).

The baseline characteristics of the 16 patients in the nintedanib prospective study are shown in Table 1. The median age was 71 years old, 88% were male, and the median %FVC predicted was 62%. Nine IPF patients received LTOT and seven IPF patients did not receive LTOT. Compared with IPF patients without LTOT, IPF patients with LTOT had significantly decreased DL_CO_ (% predicted) (*P* = 0.017) and relatively lower PaO_2_ on room air at rest, but the difference was not significant (*p* = 0.054). Among IPF patients without LTOT, none started LTOT during the study period.

**Table 1:** Baseline characteristics of patients in nintedanib prospective study

	Nintedanib	Nintedanib with LTOT	Nintedanib without LTOT	*P* value
	(*n* = 16)	(*n* = 9)	(*n* = 7)	
Age (years)	71 [57–80]	69 [57–77]	73 [64–80]	0.536
Male	14 (88%)	8 (88%)	6 (86%)	0.849
Weight (kg)	61.0 [43.3–73.9]	68.7 [43.3–73.9]	59.6 [44.3–61.5]	0.055
Body mass index	24.2 [15.1–26.9]	25.1 [15.1–26.9]	22.0 [19.0–26.7]	0.210
Smoking status				
Never / Former / Current smoker	1 / 15 / 0	0 / 9 / 0	0 / 7 / 0	–
Time since the diagnosis of IPF (years)	4 [0–9]	2 [0–9]	4 [1–8]	0.408
Long-term oxygen treatment	9 (56%)	–	–	–
mMRC dyspnea scale	2 [0–3]	3 [0–3]	1 [0–2]	0.114
GAP score	4 [2–8]	5 [2–8]	4 [3–5]	0.174
SpO_2_ on room air at rest (%)	96 [94–99]	95 [94–97]	97 [94–99]	0.234
PaO_2_ on room air at rest (mmHg)	83 [59–122]	75 [59–94]	88 [81–122]	0.054
PASP measured by TTE at baseline (mmHg)	34.0 [21.0–59.0]	33.0 [21.0–59.0]	35.0 [27.6–55.3]	1.000
Pulmonary function tests				
FVC (mL)	1.930 [1.060–3030]	2.140 [1.060–3.030]	1.840 [1.540–2.690]	0.408
FVC (% predicted)	62 [28–88]	66 [28–88]	57 [51–79]	0.758
FEV_1_ (mL)	1.700 [1.030–2.470]	1.990 [1.030–2.407]	1.560 [1.330–2.270]	0.536
FEV_1_ (% predicted)	68 [34–91]	67 [34–91]	76 [50–81]	0.758
DL_CO_ (mL/min/mmHg)	10.2 [5.1–18.6]	8.3 [5.1–13.6]	10.7 [8.1–18.6]	0.209
DL_CO_ (% predicted)	70 [30–91]	49 [30–76]	70 [48–91]	0.017
Laboratory findings				
BNP	31.6 [7.6–1.313]	28.4 [7.6–1.313]	27.2 [10.0–53.9]	0.837
Radiographic findings				
Pulmonary emphysema	6 (38%)	5 (56%)	1 (14%)	0.121
Comorbidity				
Hypertension	6 (38%)	3 (33%)	3 (43%)	0.696
Chronic heart failure	0 (0%)	0 (0%)	0 (0%)	–

Data are presented as median [range] or number of patients (percentage)

*IPF* idiopathic pulmonary fibrosis, *LTOT* long-term oxygen treatment, *mMRC* modified Medical Research Council, *GAP* Gender-Age-Physiology, *SpO*_*2*_ arterial oxygen saturation measured by pulse oximetry, *PaO*_*2*_ partial pressure of arterial oxygen, *PASP* pulmonary artery systolic pressure, *TTE* transthoracic echocardiography, *FVC* forced vital capacity, *FEV*_*1*_ forced expiratory volume in 1 s, *DL*_*CO*_ diffusing capacity of the lung for carbon monoxide, *BNP* brain natriuretic peptide

*P* value: nintedanib with LTOT vs nintedanib without LTOT

**Fig. 2:**
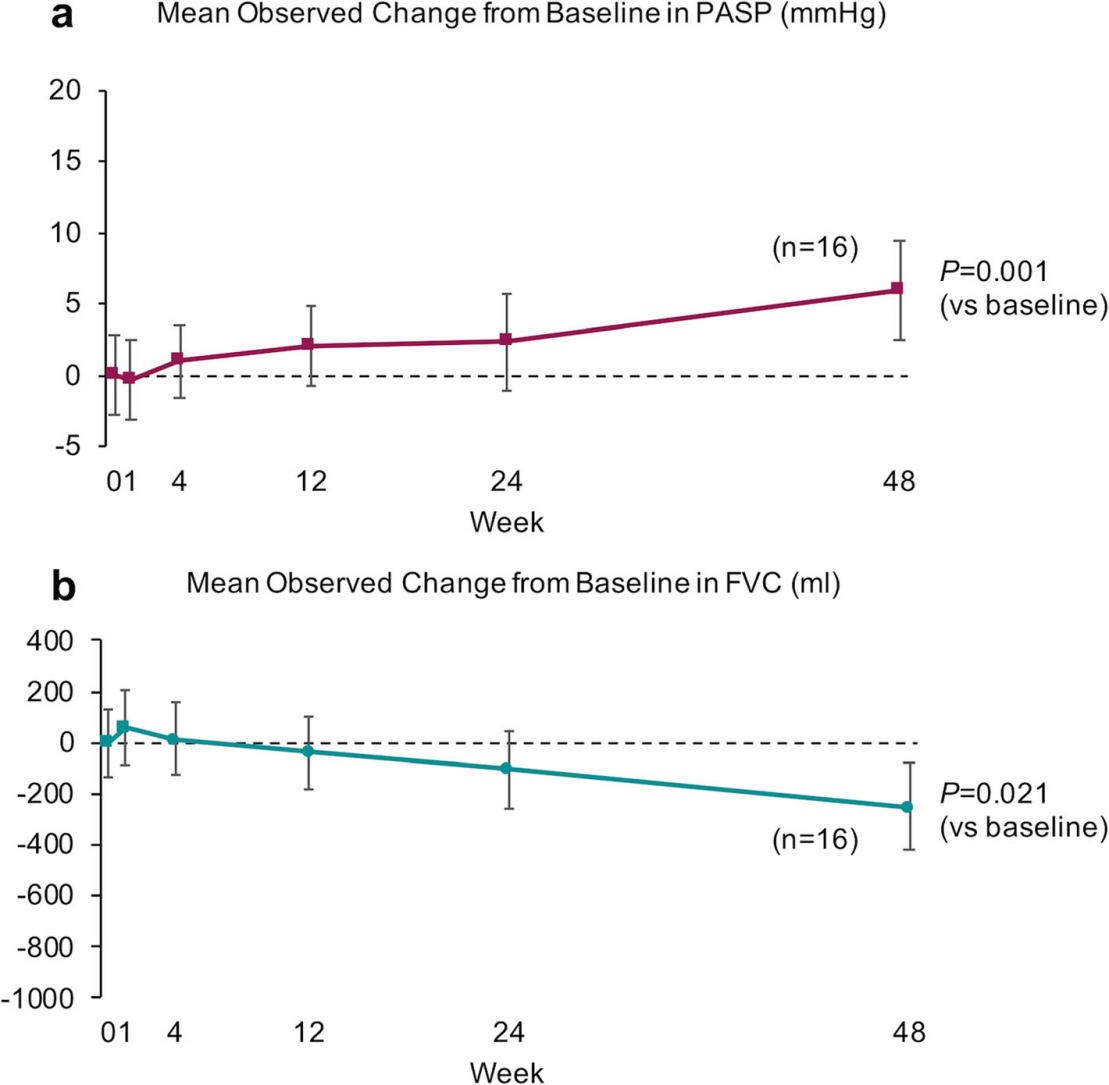
Mean observed change from baseline in PASP and FVC in nintedanib prospective study. **a** The mean observed change from baseline in pulmonary artery systolic pressure (PASP) after starting nintedanib treatment. The value of the mean changes in PASP gradually increased and PASP was significantly higher at 48 weeks after nintedanib treatment than at baseline (*P* = 0.001). **b** The mean value of changes in forced vital capacity (FVC) gradually decreased and were significantly lower at 48 weeks after nintedanib treatment than at baseline (*P* = 0.021). Changes in mean PASP and FVC from baseline to 48 weeks after treatment were assessed using a paired t-test. Two-sided *P*-values  0.05 were considered significant

**Fig. 3:**
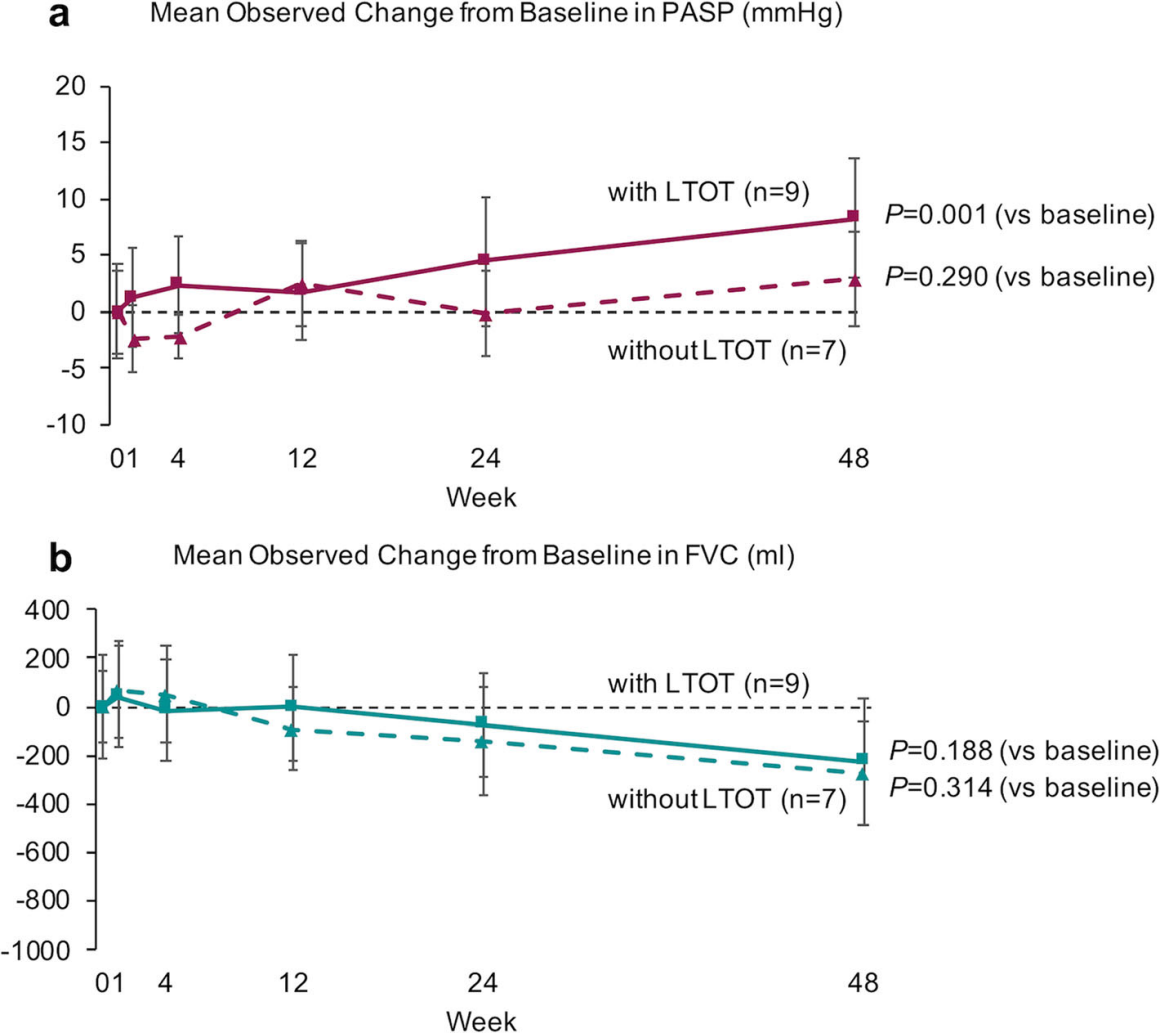
Mean observed change in PASP and FVC between IPF patients with LTOT and without LTOT. **a** The values of mean pulmonary artery systolic pressure (PASP) changes after nintedanib treatment in idiopathic pulmonary fibrosis (IPF) patients with (solid line) and without (dotted line) long-term oxygen treatment (LTOT). Mean PASP increases were 8.24 mmHg and 2.84 mmHg, respectively. There was a significant increase in the value of the mean PASP 48 weeks after nintedanib relative to baseline in IPF patients with LTOT (*P* = 0.001). **b** The values of mean forced vital capacity (FVC) changes after nintedanib treatment in IPF patients with (solid line) and without (dotted line) LTOT. mean FVC decreases were 224 mL and 270 mL, respectively. There were no significant decreases in the mean FVC relative to baseline in either group (*P* = 0.188 and *P* = 0.314). Changes in the value of the mean PASP and FVC from baseline to 48 weeks after nintedanib treatment were assessed using a paired t-test. Two-sided *P*-values < 0.05 were considered significant

Figure 2 shows the values of the mean changes in PASP (a) and FVC (b) from baseline in the nintedanib prospective study. The value of the mean changes in PASP gradually increased and compared to baseline, PASP was significantly higher 48 weeks after nintedanib treatment (*P* = 0.001) (Fig. 2a). The mean value of changes in FVC gradually decreased and was significantly lower 48 weeks after nintedanib treatment compared with baseline (*P* = 0.021) (Fig. 2b). Figure 3 shows the difference in the values of the mean changes in PASP (a) and FVC (b) from baseline between IPF patients with (solid line) and without LTOT (dotted line). The values of the mean PASP changes 48 weeks after nintedanib treatment in IPF patients with (solid line) and without (dotted line) LTOT were 8.24 mmHg and 2.84 mmHg, respectively. A significant increase in the value of the mean PASP from baseline was observed 48 weeks after nintedanib in IPF patients with LTOT (*P* = 0.001) (Fig. 3a). There were no significant decreases in the value of mean FVC compared to baseline in either group (*P* = 0.188 and *P* = 0.314) (Fig. 3b).

### The historical control study

Figure 1b demonstrates the patient flow schematics of the historical control study. A total of 35 consecutive IPF patients were retrospectively screened, and 20 IPF patients were excluded. Finally, 15 IPF patients without any antifibrotic medications were evaluated (Fig. 1b).

**Fig. 4:**
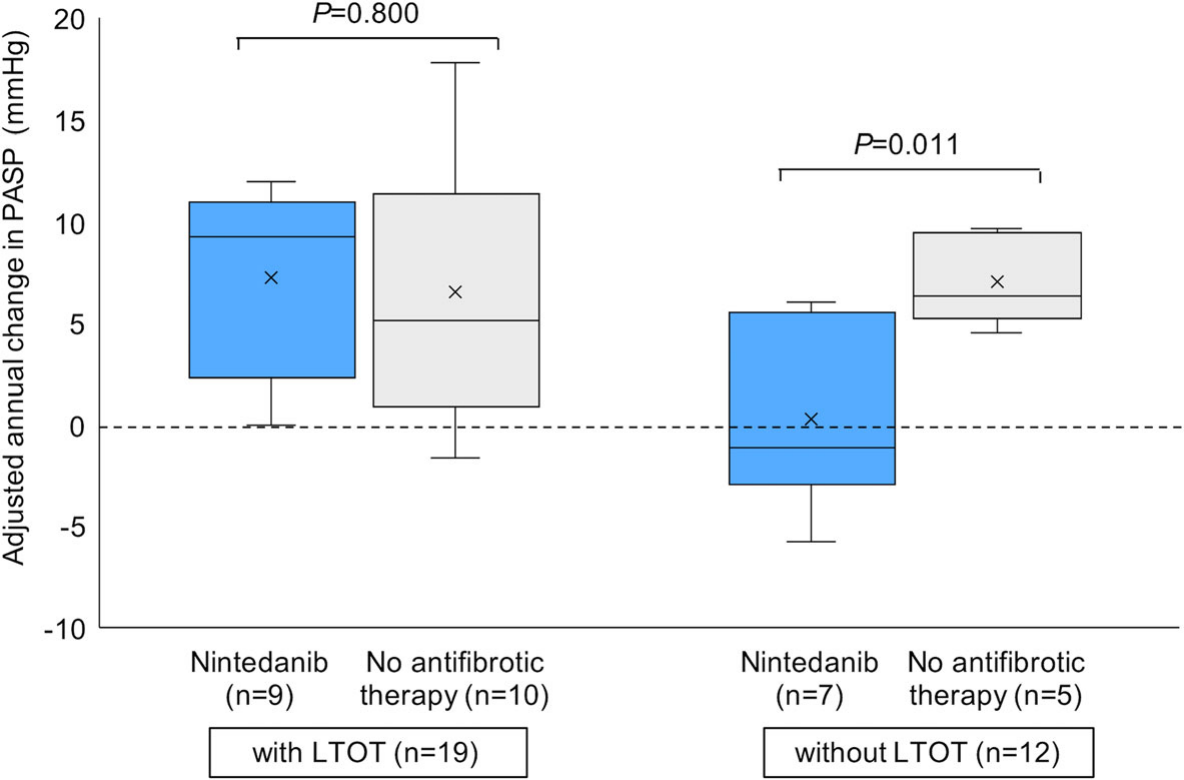
Comparison of adjusted annual change in PASP in historical control study. Comparison of adjusted annual changes in pulmonary artery systolic pressure (PASP) between the nintedanib groups and no antifibrotic therapy groups in idiopathic pulmonary fibrosis (IPF) patients with long-term oxygen treatment (LTOT) (left) and without LTOT (right). There were no significant differences between the nintedanib and no antifibrotic therapy groups (7.19 mmHg vs 6.48 mmHg; *P* = 0.800) in IPF patients with LTOT. Conversely, among IPF patients without LTOT, the adjusted annual change in PASP was significantly lower in the nintedanib group than in the no antifibrotic therapy group (0.26 mmHg vs 7.05 mmHg; *P* = 0.011). Adjusted annual change in PASP was assessed using a two-sample t-test. Two-sided *P*-values < 0.05 were considered significant

Table 2 shows the baseline characteristics of patients treated with nintedanib (the nintedanib group) and those treated with any antifibrotic therapy (the no antifibrotic therapy group) in the historical control study. Among IPF patients with LTOT, the nintedanib group consisted of nine patients and 10 patients were categorized in the no antifibrotic therapy group. Regarding the IPF patients without LTOT, seven patients were in the nintedanib group and five patients were in the no antifibrotic therapy group. Among IPF patients with and without LTOT, there were no significant differences in characteristics between the nintedanib group and no antifibrotic therapy group. Figure 4 shows the comparison of adjusted annual change in PASP between the nintedanib and no antifibrotic therapy groups in IPF patients with and without LTOT. There were no significant differences between the nintedanib and no antifibrotic therapy groups (7.19 mmHg vs 6.48 mmHg; *P* = 0.800) in IPF patients with LTOT. Conversely, among IPF patients without LTOT, the adjusted annual change in PASP was significantly lower in the nintedanib group than in the no antifibrotic therapy group (0.26 mmHg vs 7.05 mmHg; *P* = 0.011). There were no significant differences in the adjusted annual change in FVC between the nintedanib and no antifibrotic therapy groups in IPF patients with and without LTOT (see Additional file 1).

**Table 2:** Baseline characteristics between the nintedanib group and no antifibrotic therapy group in historical control study

	IPF patients with LTOT (*n* = 19)		*P* value	IPF patients without LTOT (n = 12)		*P* value
	Nintedanib	No antifibrotic therapy		Nintedanb	No antifibrotic therapy	
	(*n* = 9)	(*n* = 10)		(*n* = 7)	(*n* = 5)	
Age (years)	69 [57–77]	72 [60–83]	0.315	73 [64–80]	70 [48–76]	0.639
Male	8 (88%)	6 (60%)	0.153	6 (86%)	4 (80%)	0.793
Weight (kg)	68.7 [43.3–73.9]	54.7 [26.0–72.0]	0.065	59.6 [44.3–61.5]	52.9 [46.9–63.0]	0.876
Body mass index	25.1 [15.1–26.9]	20.2 [13.7–28.8]	0.661	22.0 [19.0–26.7]	20.7 [18.6–26.7]	0.876
Smoking status						
Never / Former / Current smoker	0 / 9 / 0	3 / 7 / 0	–	0 / 7 / 0	1 / 4 / 0	–
Time since the diagnosis of IPF (years)	2 [0–9]	1 [0–3]	0.400	4 [1–8]	3 [0–4]	0.202
mMRC dyspnea scale	3 [0–3]	2 [1–3]	0.842	1 [0–2]	2 [0–3]	0.343
GAP score	5 [2–8]	5 [1–7]	0.796	4 [3–5]	5 [2–5]	0.648
SpO_2_ on room air at rest (%)	95 [94–97]	95 [89–98]	0.573	97 [94–99]	96 [95–98]	0.648
PaO_2_ on room air at rest (mmHg)	75 [59–94]	70 [47–89]	0.277	88 [81–122]	91 [75–103]	0.927
PASP measured by TTE at baseline (mmHg)	33.0 [21.0–59.0]	41.4 [31–56.2]	0.278	35.0 [27.6–55.3]	37.0 [27.9–56.2]	0.775
Pulmonary function tests						
FVC (mL)	2.140 [1.060–3.030]	1.735 [1.170–2.480]	0.278	1.840 [1.540–2.690]	2.020 [1.060–2.370]	0.953
FVC (% predicted)	66 [28–88]	61 [46–74]	0.720	57 [51–79]	64 [35–73]	0.530
FEV_1_ (mL)	1.990 [1.030–2.407]	1.560 [1.010–2.280]	0.356	1.560 [1.330–2.270]	1.930 [1.050–2.100]	1.000
FEV_1_ (% predicted)	67 [34–91]	72 [56–83]	0.549	76 [50–81]	73 [40–85]	0.876
DL_CO_ (mL/min/mmHg)	8.3 [5.1–13.6]	6.6 [4.3–11.7]	0.091	10.7 [8.1–18.6]	10.0 [7.3–16.3]	0.788
DL_CO_ (% predicted)	49 [30–76]	40 [29–73]	0.299	70 [48–91]	74 [38–129]	1.000
Radiographic findings						
Pulmonary emphysema	5 (56%)	3 (30%)	0.414	1 (14%)	1 (20%)	0.682
Comorbidity						
Hypertension	3 (33%)	4 (40%)	0.764	3 (43%)	1 (20%)	0.408
Chronic heart failure	0 (0%)	0 (0%)	–	0 (0%)	0 (0%)	–
No antifibrotic agent						
No treatment	–	3 (30%)	–	–	1 (20%)	–
Corticosteroid monotherapy	–	1 (10%)	–	–	2 (40%)	–
Combined corticosteroid and immunomodulator therapy	–	5 (50%)	–	–	2 (40%)	–
Inhalation of N-acetyl-cysteine	–	1 (10%)	–	–	0 (0%)	–

Data are presented as median [range] or number of patients (percentage)

*IPF* idiopathic pulmonary fibrosis, *LTOT* long-term oxygen treatment, *mMRC* modified Medical Research Council, *GAP* Gender-Age-Physiology, *SpO*_*2*_ arterial oxygen saturation measured by pulse oximetry, *PaO*_*2*_ partial pressure of arterial oxygen, *PASP* pulmonary artery systolic pressure, *TTE* transthoracic echocardiography, *FVC* forced vital capacity, *FEV*_*1*_ forced expiratory volume in 1 s, *DL*_*CO*_ diffusing capacity of the lung for carbon monoxide

*P* value: the nintedanib group vs the no antifibrotic therapy group

## Discussion

This study is the first prospective echocardiographic assessment of PH after starting nintedanib treatment in IPF patients with or without LTOT. Our prospective study compared the effects of nintedanib in IPF patients with or without LTOT and showed that PASP at 48 weeks after starting nintedanib in IPF patients with LTOT was significantly increased, but not in IPF patients without LTOT (Fig. 3a). Additionally, our retrospective study demonstrated that adjusted annual change of PASP in IPF patients without LTOT treated with nintedanib was unchanged or decreased but adjusted annual change of PASP in IPF patients with LTOT treated with nintedanib was similar to that in patients without antifibrotic agents (Fig. 4). Collectively, nintedanib might cause deterioration of PH in hypoxic IPF patients; conversely, nintedanib may prevent PH in normoxic IPF patients. Our results may suggest that physicians should be aware of a deterioration in PH after starting nintedanib in IPF patients with LTOT or hypoxic IPF patients. In addition, nintedanib treatment may possibly be beneficial in IPF patients before developing severe respiratory failure.

Adverse effects of nintedanib on PH in hypoxic IPF patients may partly be explained by the finding that exacerbation of severe PH was observed in hypoxic mice treated with a selective inhibitor of VEGF-2 receptor (SU5416) in comparison with hypoxic control mice without SU5416 treatment [7]. At least as a VEGF-2 receptor inhibitor, nintedanib treatment in hypoxic IPF patients may possibly lead to similar consequences as seen in this hypoxic animal model. Several studies have reported poor adherence to oxygen therapy in chronic respiratory diseases [13, 14], and patients with LTOT may frequently be exposed to hypoxia during exercise. Similarly, IPF patients with LTOT may frequently be exposed to hypoxia in our study. Therefore, treating hypoxic IPF patients with nintedanib might provoke PH in IPF patients with LTOT. In fact, Shimomura et al. have reported PH can be exacerbated by nintedanib administration in IPF patients with comorbid chronic hypoxia [15].

The protective effects of nintedanib on PH in normoxic IPF patients have been unclear so far, but a recent report evaluating the effect of nintedanib on the proliferation of human pulmonary microvascular endothelial cells (MVEC) in a rat PH model showed that nintedanib itself might have protective effects against right ventricular hypertrophy [16]. In this report, VEGF-induced proliferation of control MVECs obtained from control patients was reduced by nintedanib, and nintedanib inhibited VEGF-stimulated phosphorylation of ERK1/2 in a dose dependent manner [16]. In an SU5416-induced PH rat model that was firstly exposed to 10% hypoxia for four weeks and then four weeks of exposure to normoxia, subsequent normoxic nintedanib treatment demonstrated less dilatation, and decreased fibrosis and hypertrophy with decreased collagen deposition in the right ventricle [16]. This could be partly explained by the reduced fibronectin production of cardiac fibroblasts exposed to nintedanib [16]. In addition, the prophylactic effects of nintedanib on the natural progression of pulmonary fibrosis may also prevent deterioration of PH in IPF patients, and collectively, nintedanib may prevent exacerbation of PH in normoxic IPF patients. Future investigations are needed to reveal the comprehensive mechanisms of the effects of nintedanib on PH.

Several limitations of this study should be addressed. First, this was a single-centre study with a relatively small sample size. Second, the nintedanib prospective study only included patients treated with nintedanib, and no control arm was conducted. However, a historical control was used as a control arm similar to a previous study [17]. In addition, we regularly perform TTE at least once a year to detect the early onset and deterioration of PH in all patients with interstitial pneumonia, including IPF. Therefore, we think that this selection bias of performing echocardiography in the historical control group may be minimal. Third, we could not evaluate right heart catheter testing. Fourth, the tricuspid annular plane systolic excursion, tissue doppler imaging systolic wall motion velocity, and Tei index in echocardiographic assessment were not included because high-quality RV-focused images were not obtained from IPF patients in our study.

## Conclusions

We examined the differential effects of nintedanib on PH between IPF patients with or without LTOT in this temporal echocardiographic assessment. Nintedanib may have a disadvantageous effect on PH in IPF patients with LTOT. Conversely, nintedanib treatment may also be beneficial to PH in IPF patients without LTOT. Accordingly, early nintedanib treatment might be more beneficial than starting with hypoxia in IPF patients with respiratory failure. Prospective randomized placebo-controlled trials are expected to elucidate proper nintedanib treatment in terms of protecting progression of PH in IPF patients.

## Additional file

Additional file 1: Comparison of adjusted annual change in FVC in historical control study. Comparison of adjusted annual changes in forced vital capacity (FVC) between the nintedanib group and no antifibrotic therapy group in idiopathic pulmonary fibrosis (IPF) patients with long-term oxygen treatment (LTOT) (left) and without LTOT (right). There were no significant differences in the adjusted annual change between the nintedanib and no antifibrotic therapy groups in IPF patients with and without LTOT. Adjusted annual change in FVC was assessed using a two-sample t-test. Two-sided *P* values of < 0.05 were considered significant. (TIFF 9067 kb)

## Data Availability

The datasets used for the current study are available from the corresponding author on reasonable request.

## Abbreviations

FGF: Fibroblast growth factor
FVC: Forced vital capacity
GAP: The Gender-Age-Physiology
IPF: Idiopathic pulmonary fibrosis
LTOT: Long-term oxygen treatment
mMRC: Modified Medical Research Council
MVEC: Microvascular endothelial cells
PASP: Pulmonary artery systolic pressure
PDGF: Platelet-derived growth factor
PH: Pulmonary hypertension
RAP: Right atrial pressure
TR: Tricuspid regurgitation
TTE: Transthoracic echocardiography
VEGF: Vascular endothelial growth factor
