# Supplementary figures and images for: Temporal echocardiographic assessment of pulmonary hypertension in idiopathic pulmonary fibrosis patients treated with nintedanib with or without oxygen therapy
Source: BMC Pulm Med. 2019 Aug 22;19:157. doi: 10.1186/s12890-019-0918-3 (PMC6704493; doi:10.1186/s12890-019-0918-3)

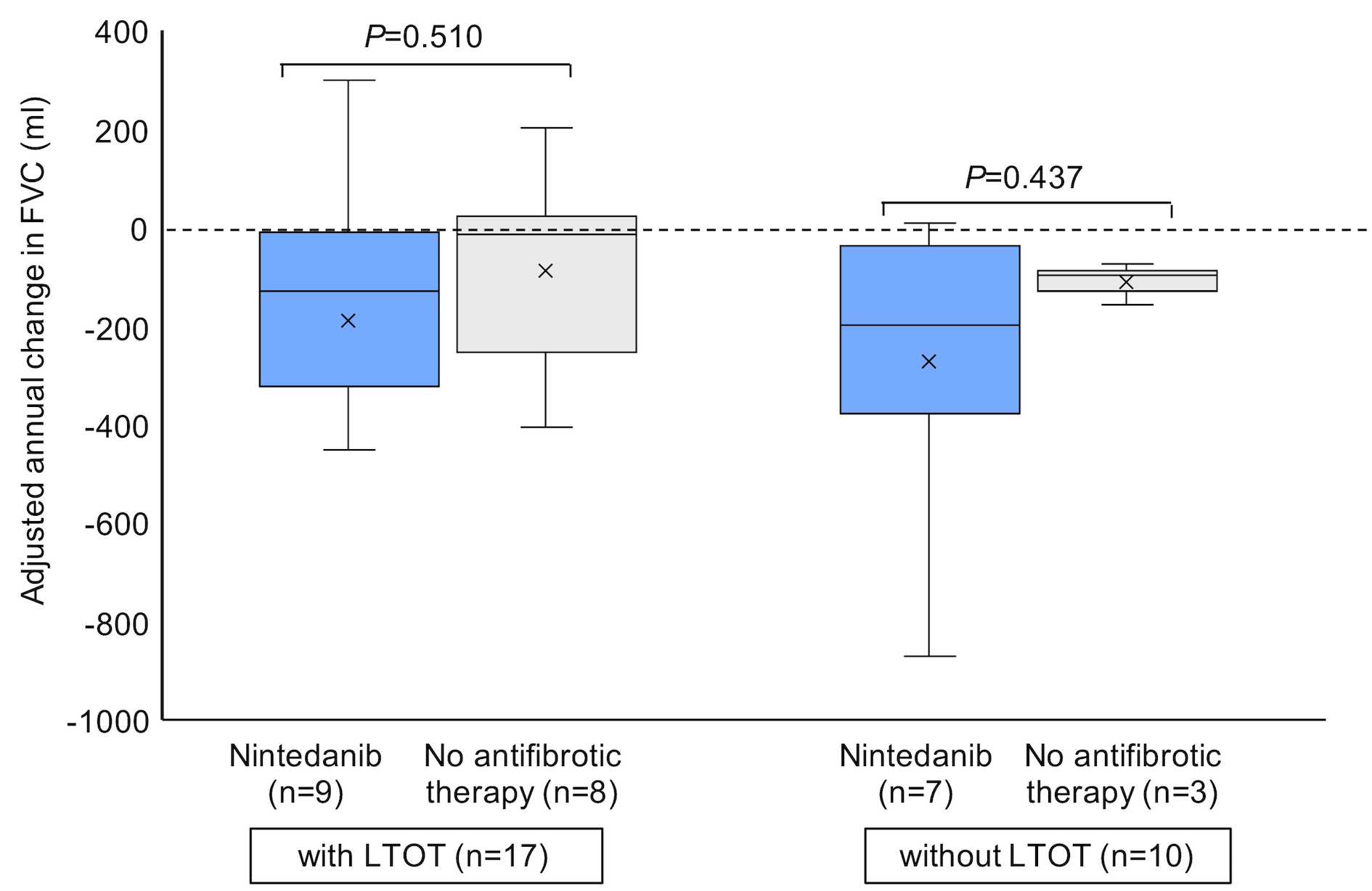

Supplement: Supplementary file 1 — Comparison of adjusted annual change in FVC in historical control study. Comparison of adjusted annual changes in forced vital capacity (FVC) between the nintedanib group and no antifibrotic therapy group in idiopathic pulmonary fibrosis (IPF) patients with long-term oxygen treatment (LTOT) (left) and without LTOT (right). There were no significant differences in the adjusted annual change between the nintedanib and no antifibrotic therapy groups in IPF patients with and without LTOT. Adjusted annual change in FVC was assessed using a two-sample t-test. Two-sided P values of < 0.05 were considered significant. (TIFF 9067 kb) [file 12890_2019_918_MOESM1_ESM.tiff]
